# Potential Therapeutic Applications of Hydrogen in Chronic Inflammatory Diseases: Possible Inhibiting Role on Mitochondrial Stress

**DOI:** 10.3390/ijms22052549

**Published:** 2021-03-04

**Authors:** Shin-ichi Hirano, Yusuke Ichikawa, Bunpei Sato, Haru Yamamoto, Yoshiyasu Takefuji, Fumitake Satoh

**Affiliations:** 1 Department of Research and Development, MiZ Company Limited, 2-19-15 Ofuna, Kamakura, Kanagawa 247-0056, Japan; s_hirano@e-miz.co.jp (S.-i.H.); y_ichikawa@e-miz.co.jp (Y.I.); b_sato@e-miz.co.jp (B.S.); info@e-miz.co.jp (F.S.); 2 Department of Molecular & Cell Biology, University of California, 3060 Valley Life Sciences Bldg #3140, Berkeley, CA 94720-3140, USA; haru.yamamoto@berkeley.edu; 3 Faculty of Environment and Information Studies, Keio University, 5322 Endo, Fujisawa 252-0882, Japan

**Keywords:** hydrogen, chronic inflammatory disease, medical application, hydroxyl radical, reactive oxygen species, NLRP3, COVID-19

## Abstract

Mitochondria are the largest source of reactive oxygen species (ROS) and are intracellular organelles that produce large amounts of the most potent hydroxyl radical (**·**OH). Molecular hydrogen (H_2_) can selectively eliminate **·**OH generated inside of the mitochondria. Inflammation is induced by the release of proinflammatory cytokines produced by macrophages and neutrophils. However, an uncontrolled or exaggerated response often occurs, resulting in severe inflammation that can lead to acute or chronic inflammatory diseases. Recent studies have reported that ROS activate NLRP3 inflammasomes, and that this stimulation triggers the production of proinflammatory cytokines. It has been shown in literature that H_2_ can be based on the mechanisms that inhibit mitochondrial ROS. However, the ability for H_2_ to inhibit NLRP3 inflammasome activation via mitochondrial oxidation is poorly understood. In this review, we hypothesize a possible mechanism by which H_2_ inhibits mitochondrial oxidation. Medical applications of H_2_ may solve the problem of many chronic inflammation-based diseases, including coronavirus disease 2019 (COVID-19).

## 1. Introduction

Inflammation is induced by the release of proinflammatory cytokines, such as interleukin (IL)-1β and IL-18, produced by macrophages and neutrophils in direct response to a triggering stimulus. Normally, the production of these cytokines is transient; however, if the cytokines are continuously produced by some disturbance, the inflammation can be delayed and chronic inflammation may develop. Minor but prolonged inflammation can damage the living body and induce chronic inflammation. Recent studies have shown that mitochondria play a key role in producing IL-1β and IL-18 [1]. It has also been reported that mitochondrial reactive oxygen species (ROS) activate the nucleotide-binding and oligomerization domain-like receptor family pyrin, which contains a complex of intracellular proteins known as 3 (NLRP3) inflammasome, and that its stimulation triggers the production of inflammatory cytokines [2,3,4,5,6,7,8,9,10,11].

The mitochondrial selective hydroxyl radical (·OH) scavenger may block the cascade leading to the activation of the NLRP3 inflammasome in the human body, because a large amount of ·OH can be produced in the mitochondria [12]. Modern medical treatment can control acute inflammatory diseases, but it cannot control chronic inflammatory diseases. Attempts have been made to develop effective therapeutics to control chronic inflammatory diseases, but their development strategies have been unsatisfactory, because they are not based on the underlying mechanisms of chronic inflammatory diseases.

Molecular hydrogen (H_2_) is an antioxidant that can selectively scavenge ·OH, which is the most potent oxidant among ROS [12]. Among the homonuclear diatomic molecules (N_2_, O_2_, etc.) that can permeate the cell membrane, only H_2_ is able to scavenge ·OH, which is always generated inside of mitochondria [13,14]. It has been shown by literature that H_2_ in various animal models of inflammation may be based on mechanisms to inhibit mitochondrial oxidation and NLRP3 inflammasome activation [15,16,17,18,19,20,21,22,23]. However, the ability for H_2_ to inhibit mitochondrial oxidation and NLRP3 inflammasome activation is poorly understood. In this review, we will propose a possible mechanism by which H_2_ inhibits NLRP3 activation via the inhibition of mitochondrial oxidation and then present a perspective on the potential effects of H_2_ on chronic inflammatory diseases including coronavirus disease 2019 (COVID-19). Furthermore, since senescence is a part of chronic inflammation, we will mention the potential of H_2_ to control senescence.

## 2. Mitochondrial Roles in the Regulation of Inflammation

Inflammation is considered to be one of the immediate responses of the innate immune system. Inflammation is triggered by the invasion of pathogens or by mechanical damage to cells and tissues. These responses are recognized by pattern recognition receptors (PRRs), which include toll-like receptors (TLRs) attached to the cell membrane or nucleotide-binding oligomerization domain-like receptors (NLRs) presented in the cytoplasm [24]. Pathogen-associated molecular patterns (PAMPs) are recognized by PRRs in infections. In the presence of mechanical damage, damage-associated molecular patterns (DAMPs) are also recognized by PRRs. After PAMPs or DAMPs are recognized by PRRs, innate immune responses are initiated [25]. These responses utilize innate immune cells such as macrophages, neutrophils, and proinflammatory cytokines, and the responses can be considered as a regulated host defense mechanism. However, on rare occasions when uncontrolled or excessive responses occur, the result is severe inflammation, leading to acute or chronic inflammatory diseases.

Inflammasomes are multiprotein complexes that play a role in signal transduction. Inflammasomes play an important role in mediating innate inflammatory responses; they assemble in response to a variety of stimuli, including PAMPs and DAMPs. Among inflammasomes, NLRP3 has been extensively studied and characterized because of its critical role in immunity and inflammation [26,27]. The major components of this signaling platform include the NLRP3 protein, its adaptor protein apoptosis-associated speck-like proteins containing a CARD (ASC), and caspase-1, which is cleaved by this multiprotein complex. The cleavage of procaspase-1 cleaves the proinflammatory cytokines, such as IL-1β and IL-18, into their active forms, producing active caspase-1, a proteolytic enzyme that induces an inflammatory response.

The activity of NLRP3 is strongly inhibited by ubiquitination under normal conditions. However, when the cells are stimulated with lipopolysaccharide (LPS) or ATP, deubiquitination of NLRP3 is induced. The NLRP3 induces the activation of proinflammatory cytokines by releasing this suppression [28]. LPS and ATP induce the production of proinflammatory cytokines via transmembrane TLR4 and P2X purinoceptor 7 (P2 × 7) receptors, respectively. The process of this induction is dependent on the production of mitochondrial reactive oxygen species (mtROS). Furthermore, oxidized mitochondrial DNA (mtDNA), an oxidation product of ROS released from mitochondria, has been reported to bind and activate NLRP3 [6]. Mitochondria-targeted antioxidants may inhibit NLRP3-induced activation of inflammatory cells by inhibiting the production of oxidized mtDNA. In addition, recent studies have identified cytidine/uridine monophosphate kinase 2 (CMPK2) as a molecule required to induce gene expression for the activation of NLRP3 inflammasome, through stimulations with molecules such as LPS [29].

## 3. H_2_ as a Mitochondria-Targeted ·OH Scavenger

H_2_ is a flammable, colorless, odorless, and non-toxic gas. The first report on the therapeutic effect of H_2_ was published by Dole et al. in 1975 [30]. They demonstrated that hyperbaric treatment of 2.5% oxygen gas and 97.5% H_2_ gas could markedly induce tumor regression in mice. However, research on medical applications of H_2_ was not extensively conducted except for some pioneering studies. In 2007, Ohsawa et al. reported that H_2_ ameliorated ischemia-reperfusion injuries in a rat model with cerebral infarction [12]. They demonstrated that H_2_ selectively scavenges two types of potent ROS, namely ·OH and peroxynitrite (ONOO-). However, we cannot overlook the pioneering study by Yanagihara et al. in 2005, two years before Ohsawa’s study. They reported that the intake of neutral H_2_-rich water produced by electrolysis can effectively reduce oxidative stress caused by chemical oxidants in rats [31]. H_2_ has been characterized as a potent antioxidant capable of attenuating oxidative stress-related diseases [32,33]. H_2_ has remarkable therapeutic effects on various diseases, including cancer [34], sepsis [35], cardiovascular disease [36], neuronal disease [37], diabetes [38], metabolic syndrome [39], etc. Up until now, more than 800 original papers regarding the medical use of H_2_, including approximately 70 clinical trials, have been reported. As observed from the production of H_2_ in the intestines, adverse effects caused by H_2_ has not been observed in many clinical studies [40,41,42,43,44,45,46]. In recent papers, we reviewed that H_2_ is promising for medical applications due to its marked efficacy and for having no adverse effects [47].

Vitamins, known as antioxidants, are unable to scavenge ·OH generated inside of mitochondria because they have difficulty penetrating cell membranes and reaching intracellular organelles such as mitochondria. Homonuclear diatomic molecules that can exist in living bodies are nitrogen molecule (N_2_), oxygen molecule (O_2_), H_2_, and halogens (Cl_2_, I_2_, etc.). Among these, O_2_ is converted to energy in living bodies and is used for oxidation and hydroxylation of biological materials by oxygenating enzymes. Halogens in general are extremely active and act as poisons to the living body. On the other hand, N_2_ is an inactive molecule because it does not react with substances constituting of cells and there is no metabolic system that metabolizes the N_2_.

H_2_ is also an inactive molecule that has no metabolic system in mammalian cells and does not interact with biological substances, but it is a molecule that can react with ·OH, which are generated inside mitochondria [12]. In addition, because H_2_ itself is an inert substance and the reaction product of H_2_ and ·OH is a water molecule, it does not have any adverse effects on the living body, unlike drugs. In a recent paper, we proposed that H_2_ is the only molecule that can enter mitochondria and undergo a hydrogen withdrawal reaction with ·OH [13,14]. Thus, H_2_ is the only diatomic molecule that can enter mitochondria to protect cells from cytotoxicity caused by ·OH. Since H_2_ is a diatomic molecule essential for the survival of life, we proposed in the paper that H_2_ is a “philosophical molecule” [14].

## 4. Effects of H_2_ on Inflammation and Its Mechanisms

### 4.1. Possible Mechanisms for Targeting mtROS Production and NLRP3 Activation by H_2_

It has been shown that the NLRP3 inflammasome has been linked to disease such as atherosclerosis, acute and chronic respiratory diseases, Alzheimer’s disease, traumatic brain injury, acute kidney injury (AKI), chronic kidney disease (CKD), and cancer [4]. NLRP3 inflammasome-dependent inflammatory responses are known to be triggered by a variety of signals that endanger the host, including infection, tissue damage, and metabolic abnormalities [1]. Recent paper suggest that mitochondria integrate these different signals and relay this information to NLRP3 inflammasomes [1]. Dysfunctional mitochondria generate ROS, which in turn activate the inflammasomes.

In contrast, NLRP3 inflammasomes are negatively regulated by autophagy, which is a catabolic process that removes damaged or dysfunctional organelles including mitochondria. Activation of the inflammasome and IL-18 signaling pathway is largely protected in colitis-associated colorectal cancer. However, excessive inflammation driven by the inflammasome and IL-1β signaling pathways promotes breast cancer, fibrosarcoma, gastric cancer, and lung metastasis in a context-dependent manner [48]. Some literature has shown that H_2_ can be involved in various models of inflammation based on mechanisms of mitochondrial oxidation inhibition and NLRP3 inflammasome activation (Table 1) [15,16,17,18,19,20,21,22,23]. 

Recently, it has been reported that the production of mtROS in LPS-stimulated macrophages trigger the activation of NLRP3, but this production can be suppressed by mitochondria-targeted antioxidants. Ren et al. examined the effects of H_2_ on the activation of NLRP3 inflammasomes in in vitro experiments using mouse RAW264.7 cells [15]. They reported that the stimulation of these cells with LPS increased the expression of NLRP3, caspase-1, and IL-1β with the production of mtROS in the absence of H_2_, but the treatment of H_2_ suppressed the production of mtROS and the expression of NLRP3, caspase-1, and IL-1β. In addition, they reported that the elimination of mtROS by H_2_ in response to LPS stimulation consequently inhibited the mtROS-mediated NLRP3 deubiquitination, a non-transcriptional signal [15]. These results report a possible mechanism by which H_2_ inhibits the mtROS-mediated activation of NLRP3 production.

It has been shown that ROS induce oxidative stress and that this stress is one of the important etiological factors of acute pancreatitis. Ren et al. also examined the effects of intraperitoneal injection of H_2_-rich saline on the activation of NLRP3 inflammasome in a mouse model with acute pancreatitis [16]. The results showed that in the control group, NLRP3 inflammasome activation, increased nuclear factor-κB (NF-κB) activity, and increased production of inflammatory cytokines such as TNF-α and IL-1β were observed, but these changes were significantly suppressed by H_2_ -rich saline administration. In addition, an increase in malondialdehyde (MDA) levels and a decrease in superoxide dismutase (SOD) activity were also observed in the control group, whereas the administration of H_2_-rich saline caused a decrease in MDA levels and an increase in SOD activity. Histopathological examination of the pancreas also confirmed the efficacy of H_2_ -rich saline administration. They reported that the administration of H_2_-rich saline in acute pancreatitis contributed to the inhibition of NLRP3 inflammasome activation [16].

Ischemia/reperfusion (I/R) disorders of the intestine often lead to inflammatory reactions and coagulopathy. H_2_ exhibits anti-inflammatory, antioxidant, and anti-apoptotic effects, but its efficacy on intestinal I/R has been unclear. Yang et al. investigated the effects of intravenous administration of H_2_-rich saline on intestinal I/R in an experiment on rats [17]. They reported that the administration of H_2_-rich saline improved the survival rate of the rats and ameliorated intestinal damage, edema, and apoptosis. In addition, in this paper, they reported that the administration of H_2_-rich saline improved inflammatory response and also markedly ameliorated I/R-mediated coagulopathy in the intestine. They also reported that H_2_-rich saline inhibited the activation of NF-*κ*B and NLRP3 inflammasomes in peripheral blood mononuclear cells [17]. They suggested that the improvement of coagulopathy and inflammation by administration of H_2_-rich saline was partly due to the NF-*κ*B/NLRP3 pathway.

Limb I/R is a clinical condition that is associated with high morbidity and mortality. Zou et al. investigated the efficacy and mechanism of H_2_-rich saline on acute lung injury in rats induced by Limb I/R [18]. H_2_-rich saline was administered intraperitoneally when the clips of limbs were released. The results showed that H_2_-rich saline improved survival and the edema, injury, and apoptosis in lung tissues. H_2_-rich saline also decreased levels of TNF-α, IL-6, myeloperoxidase, and MDA in blood and lung tissue, and increased the activity of SOD. In addition, the H_2_-rich saline solution dawn-regulated the protein expression of chemerin and NLRP3 in lung tissue [18]. These results indicate that the activated chemerin/NLRP3 signaling pathway is partly involved in the mechanism by which H_2_ ameliorates acute lung injury.

Neuropathic pain is a complication after spinal nerve injury, and inflammasomes are thought to be a trigger for neuropathic pain. In a previous study, Chen et al. confirmed the protective effects of H_2_ on a rat model with neuropathic pain, but the mechanism of its efficacy was unclear. Therefore, they investigated the effect of intraperitoneal administration of H_2_-rich saline on this pain model [19]. The results showed that neuropathic pain stimulated NLRP3 inflammasome activation and an autophagy pathway in spinal microglial cells. H_2_ induced autophagy-related protein expression and inhibited NLRP3 pathway activation. Furthermore, H_2_ alleviated the hyperpathia induced by neuropathic pain. They reported that H_2_ ameliorated hyperpathia through its mechanism to inactivate autophagy-mediated NLRP3 inflammasomes [19].

Early brain injury (EBI) after subarachnoid hemorrhage (SAH) occurs within 72 h and is associated with inflammation and apoptosis. Recent reports have shown that H_2_-rich saline improves EBI after SAH, but the mechanism remained unclear. Shao et al. investigated the mechanism of H_2_-rich saline on a rat model with SAH [20]. The results showed that in the control group, SAH increased the protein levels of NLRP3, ASC, caspase-1, IL-1β, and cleaved caspase-3, and the mRNA levels of IL-1β, IL-6, and TNF-α in brain tissue, but these changes were ameliorated in the H_2_ group. In addition, there was an increase in ROS and MDA and a decrease in SOD in brain tissue in the control group, but these changes were improved in the H_2_ group [20]. They reported that inactivation of the NF-κB pathway and NLRP3 inflammasome was partly involved in the mechanism by which H_2_-rich saline improved inflammation in EBI [20].

SAH is a cerebrovascular disease with poor prognosis. NLRP3 plays an important role in the inflammatory response, which may lead to vascular endothelial cell damage and disruption of the blood-brain barrier (BBB). Zhuang et al. examined the effects of H_2_ gas inhalation in a rat model of SAH [21]. SAH upregulated the expression of NLRP3 and ASC, accompanied by an increase in inflammatory and apoptotic markers. However, inhalation of H_2_ gas decreased these markers and also suppressed the expression of NLRP3 and ASC. Moreover, H_2_ gas inhalation also improved cerebral edema and vasospasm. They reported that the mechanism by which H_2_ suppresses oxidative stress-related endothelial cell injuries may partly involve the suppression of the activation of the ROS/NLRP3 axis [21].

Sepsis-associated encephalopathy (SAE) is a major cause of death, and oxidative stress, inflammation, and apoptosis have been implicated in its pathogenesis. Xie et al. investigated the mechanism of H_2_-rich saline on SAE [22]. The results showed that SAE increased the expression of NLRP3 and Nrf2 in microglia, while MCC950, an inhibitor of NLRP3, suppressed the expression of NLRP3, the release of IL-1β and IL-18, apoptosis, and mitochondrial dysfunction. H_2_-rich saline increased Nrf2 expression and inhibited SAE-induced NLRP3 expression, caspase-1, IL-1β and IL-18 release, apoptosis, and mitochondrial dysfunction in wild-type (WT) mice, while these effects of H_2_ were not observed in Nrf2 knockout (KO) mice [22]. They reported that the Nrf2-mediated NLRP3 pathway is involved in the mechanism by which H_2_ suppresses the SAE.

Sepsis is also a condition in which an organism loses control over an infection and develops lethal organ failure. Chen et al. examined the effects of H_2_-rich saline in a mouse model of sepsis induced by cecal ligation (CLP). Similarly, they also examined the effects of H_2_ treatment on macrophages induced by LPS [23]. The results showed that H_2_ treatment attenuated vital organ damage, inflammatory response, mitochondrial dysfunction, and NLRP3 pathway activation. Furthermore, H_2_ treatment induced autophagy in macrophages induced by LPS and CLP. They reported that H_2_ ameliorated mitochondrial dysfunction via autophagy-mediated NLRP3 inactivation [23].

Recently, Chen et al. demonstrated that H_2_ has neuroprotective effects on many diseases, such as neurodegenerative disease, traumatic brain injury, depression, sabarachnoid hemorrhage, and cognitive dysfunction [49]. In this review, they indicated that excessive ROS stimulate the expression of NF-κB, and promote the secretion of pro-inflammatory cytokines by activating the NLRP3 inflammasome, and that H_2_ may attenuate the inflammatory response in various CNS diseases through the inhibitory effects of NLRP3 inflammasomes. Moreover, Li et al. also reported in the review that the activation of NLRP3 inflammasomes is one of the inflammatory mechanisms in pancreatitis [50], because in the experiment by Ren et al. using acute pancreatic mice, H_2_ markedly suppressed ROS production at the source and NLRP3 inflammasome expression [16]. 

### 4.2. Possible Mechanisms by H_2_ Excluding Inhibitions of mtROS Production and NLRP3 Activation

In the previous chapter, we discussed a possible mechanism targeting mtROS production and NLRP3 activation by H_2_, but cellular pathways related to the downstream of PRRs and TLRs activation should also be considered. Many papers have reported the anti-inflammatory effects of H_2_, but very few have reported its mechanisms in detail. Some papers have discussed the possibility of NF-*κ*B, mitogen activated protein kinase (MAPK), and heme oxygenase-1 (HO-1) pathways as possible mechanisms for the anti-inflammatory effects of H_2_.

NF-κB is a transcription factor that regulates the expression of target genes such as cytokines, chemokines, adhesion molecules, and oxidative stress-related enzymes. Wang et al. investigated the effects and mechanisms of H_2_-rich saline on amyloid-β (Aβ)-induced inflammation and oxidative stress models in rats [51]. H_2_-rich saline was administered intraperitoneally after intraventricular administration of Aβ1-42 in rats. As a result, the levels of IL-1β, 8-hydroxy-2’-deoxyguanosine (8-OH-dG), c-Jun NH2-terminal kinase (JNK), and NF-*κ*B in brain tissues were increased after Aβ1-42 administration, while administration of H_2_-rich saline decreased levels of IL-1β and 8-OH-dG and activation of JNK and NF-κB [51]. They reported that the inhibitory effects of H_2_ on Aβ-induced neuroinflammation and oxidative stress were involved in the suppression of JNK and NF-κB activations.

MAPK pathway includes the extracellular-signal-regulated protein kinase (ERK), JNK, and p38 MAPK subfamilies, which function as key molecules transmitting extracellular signals to the nucleus. Liu et al. investigated the effect of intraperitoneal administration of H_2_-rich saline on hepatic injury rats with obstructive jaundice. The hepatic injury was induced by bile duct ligation [52]. The results showed that the levels of alanine aminotransferase (ALT) and aspartate aminotransferase (AST) in serum, MDA, myeloperoxidase activity, TNFα, IL-1β, IL-6, and high-mobility group 1 (HMGB1) in tissues were significantly increased, while H_2_-rich saline decreased these levels and improved histopathological liver damage [52]. In addition, H_2_-rich saline increased the activities of the antioxidant enzymes SOD and catalase, and it downregulated the activation of ERK1/2 [52]. These results suggest that the ameliorating effects of H_2_ on inflammation and oxidative stress in a rat model with liver injuries are partly due to the inhibition of the ERK1/2 pathway.

Itoh et al. stimulated mouse RAW264 cells with LPS and interferon-γ (IFN-γ) to investigate the effect of H_2_ treatment on nitric oxide (NO) production [53]. The results showed that H_2_ treatment inhibited the phosphorylation of ASK1, as well as its downstream signaling molecules, p38 MAPK and JNK. In addition, NO production was significantly suppressed by H_2_ treatment [53]. Furthermore, H_2_-rich water ameliorated type II collagen-induced arthritis in a mouse that was used as a model of human rheumatoid arthritis. They considered that H_2_ regulates macrophage signaling, and furthermore, the mechanism by which H_2_ suppresses the inflammation in mice was involved in the modification of signaling in the MAPK pathway such as with ASK, p38, and JNK [53].

Aquaporins (AQP1) and AQP5 play an important role in scavenging extravascular lung water in patients with sepsis-induced lung injury. It has been reported that H_2_-rich saline has a protective effect on sepsis-induced lung injury. Tao et al. investigated whether AQP1 and AQP5 are involved in the inhibitory effect of H_2_-rich saline on lung injuries [54]. Rats were administered with LPS intratracheally, followed by intraperitoneal administration of H_2_-rich saline. LPS significantly impaired lung function and downregulated the expression of AQP1 and AQP5, but these changes were attenuated by the administration of H_2_-rich saline. Furthermore, H_2_-rich saline suppressed LPS-induced p38 MPPK and JNK. They reported that the downregulation of AQP1 and AQP5 was associated with suppressions of p38 MAPK and JNK expressions [54].

Nonalcoholic steatohepatitis (NASH) is a disease that can progress to liver fibrosis without effective control. Li et al. investigated the efficacy and mechanism of H_2_-rich water in a mouse model with NASH [55]. The results showed that compared to the control group, the H_2_ group showed lower levels of ALT and AST and milder histological damage. In addition, H_2_-rich water inhibited liver inflammation and fibrosis, as well as apoptosis. Furthermore, in experiments using cultured hepatocytes, H_2_ treatment suppressed LPS-induced production of inflammatory cytokines through the HO-1/IL-10-independent pathway [55]. They reported that the protective effect of H_2_-rich water on hepatic injury in NASH is mediated by the HO-1/AMPK pathway [55].

### 4.3. Our Hypothesis of H_2_ on Inflammatory Disease

**Figure 1:**
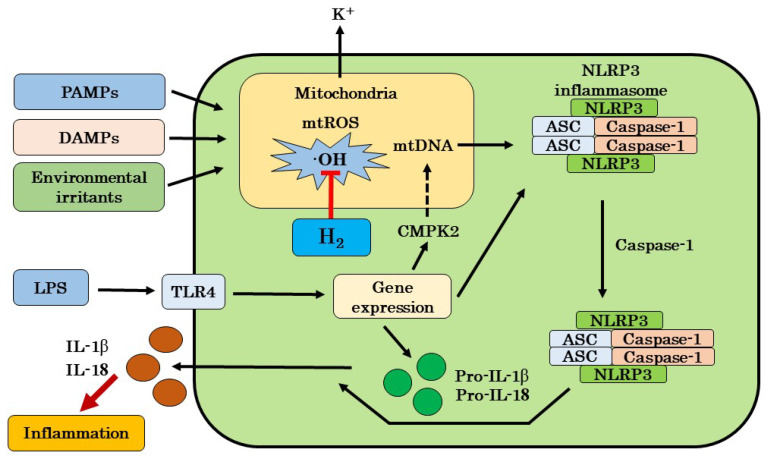
A possible mechanism of H_2_ on inflammatory diseases targeting the mtROS production and NLRP3 inflammasome activation. H_2_ can selectively eliminate **·**OH generated inside of mitochondria, and inhibit the cascade leading to NLRP3 activation by scavenging excess mtROS, and this inhibition leads to the suppression of IL-1β and IL-18 production. ROS: reactive oxygen species; **·**OH: hydroxyl radicals; H_2_: molecular hydrogen; IL-1β: interleukin-1β; IL-18: interleukin-18; NLRP3: nucleotide-binding and oligomerization domain-like receptor family pyrin domain-containing 3; TLR4: toll-like receptor 4; PAMPs: pathogen-associated molecular patters; DAMPs: damage-associated molecular patters; ASC: apoptosis-associated speck-like protein containing a CARD; LPS: lipopolysaccharide; mtROS: mitochondrial reactive oxygen species; mtDNA: mitochondrial DNA; CMPK2: cytidine/uridine monophosphate kinase 2.

Our suggested mechanism of H_2_ on chronic inflammatory diseases is that H_2_ may inhibit the cascade leading to the activation of NLRP3 by scavenging excess mtROS, and this inhibition may lead to the suppression of IL-1β and IL-18 production. However, previous papers supporting this mechanism have not shown that the ROS is **·**OH itself. The most oxidatively produced ROS in mitochondria is **·**OH [13,14]. Therefore, in this review, we hypothesized that the **·**OH scavenging effects of H_2_ may lead to the suppression of NLRP3 activation through the inhibition of mtDNA oxidation (Figure 1). In the future, it will be necessary to identify the exact ROS responsible for mitochondrial oxidation and to analyze in detail the mechanism by which H_2_ inhibits mitochondrial oxidation. 

In addition, we cannot deny the possibility that as another mechanism, H_2_ regulates cellular pathways related to the downstream of PRRs and TLRs activation. We believe that further research is needed to analyze the mechanisms of the anti-inflammatory effects of H_2_.

## 5. Possibility of H_2_ Gas Therapy for COVID-19

The epidemic caused by SARS-CoV-2, which began in Wuhan, China, in December 2019, has exploded into a worldwide pandemic, with more than 96.2 million cases of infection and 2.06 million deaths worldwide as of 22 January 2021. In the infectious disease named COVID-19, SARS-CoV-2 uses angiotensin converting enzyme 2 (ACE2) receptors as its receptor. The infection with the virus begins when a spike on the surface of the virus binds to the receptor on the surface of host cells. When the viral spike binds to its receptor (ACE2), the virus enters the intracellular vesicle. In these intracellular vesicles, the virus undergoes degradation and genetic information is released into the cytoplasm, where the virus replicates and multiplies.

The mechanism by which the replication of SARS-CoV-2 in the cytoplasm induces inflammation is not well understood, but the extrapolated theory by influenza viruses can be used [56]. The cytoplasmic components of the virus recognize TLR7 and other sensor molecules, and the mitochondria produce large amounts of ROS, including **·**OH. These ROS oxidize mtDNA, which presumably drives the cascade from NLRP3 to the release of proinflammatory cytokines. Recently, Ratajczak et al. demonstrated that the overactivation of NLRP3 induced by SARS-CoV-2 infection may be a trigger of “cytokine storm” [57]. Indeed, Rodrigues et al. indicated that the NLRP3 inflammasome is activated in patients with SARS-CoV-2 infection [58]. They found active NLRP3 inflammasome in peripheral blood mononuclear cells (PBMCs) and tissues of postmortem patients upon autopsy in patients with COVID-19. They also indicated that the inflammasome-derived products including active caspase-1 and IL-18 in the sera correlated with the markers of severity, such as IL-6 and LDH [58]. Thus, from these recent papers, H_2_ may inhibit the cascade from NLRP3 to the release of proinflammatory cytokines and thereby reduce the SARS-CoV-2-induced inflammation.

A short-term, open-label, multicenter clinical trial was conducted in China using H_2_ gas in 90 patients with COVID-19 [59]. The patients in the treatment group (44 patients) inhaled a H_2_-O_2_ gas mixture (67% H_2_, 33% O_2_), while patients in the control group (46 patients) received only standard treatment (daily O_2_ gas therapy) until discharge. The results showed that improvements in the disease severity, dyspnoea, cough, chest distress, chest pain, and oxygen saturation were greater in the treatment group than in the control group, suggesting that inhalation of H_2_ gas with oxygen gas is useful. Although the mechanisms of the ameliorating effects of H_2_ gas inhalation on COVID-19 needs further investigation, these results indicate that H_2_ gas inhalation may improve symptoms including acute and chronic inflammations caused by COVID-19.

## 6. Current Perspective for Chronic Inflammation

Chronic inflammation is at the root of many diseases. It is no exaggeration to say that “chronic inflammation is the source of all diseases”, since chronic inflammation is involved in many diseases, such as atherosclerosis, diabetes, dyslipidemia, liver cirrhosis, atopic dermatitis, asthma, rheumatoid arthritis, ulcerative colitis, Alzheimer’s disease, depression, cancer, etc. [4,5,6,7,8,9,10,11]. On the other hand, the relationship between chronic inflammation and senescence has also been studied by Arai et al. in a cohort study of more than 1,500 people with longevity [60]. They showed that inflammation markers are associated with life expectancy, and that the people with low inflammation markers tending to have a longer life expectancy than those with high inflammation markers. Thus, chronic inflammation is not only associated with diseases but also with senescence.

Modern medicine does not contribute to the fundamental treatment of diseases, because medicine is a targeted therapy that focuses on improving symptoms. In addition, with recent developments in medical technology, modern medicine can cure acute inflammatory diseases, but it is far from being able to treat chronic inflammatory diseases. Despite the fact that H_2_ is a safe medical gas, medical researchers have not looked at the medical applications of H_2_ in the past. Although many clinical and animal study papers have been published showing the efficacy of H_2_ in treating chronic inflammatory diseases, to the best of our knowledge, no review article has been published that focus on the mechanism by which H_2_ inhibits the cascade from the inhibition of mitochondrial oxidation to the activation of NLRP3 inflammasomes. The medical applications of H_2_ can solve the problem of many chronic inflammation-based diseases, including COVID-19. In addition, H_2_ may have the potential to control not only inflammatory diseases but also senescence.

## 7. Conclusions

Inflammation is induced by the release of inflammatory cytokines such as IL-1β and IL-18 produced by macrophages and neutrophils in direct response to a triggering stimulus. However, when an uncontrolled or exaggerated response occurs, the resulting severe inflammation can lead to acute or chronic inflammatory diseases. It has been reported that mitochondria-related ROS activate the NLRP3 inflammasome, and its stimulation triggers the production of these inflammatory cytokines [1]. Modern medical treatment can control acute inflammatory diseases, but it cannot control chronic inflammatory diseases.

H_2_ was found to be a potent scavenger, with no adverse effects on the human body, that selectively scavenges **·**OH, the most oxidizing ROS [12]. Mitochondrial selective **·**OH scavengers may block the cascade leading to the activation of NLRP3 inflammasomes in humans. It has been shown by literature that H_2_ can be in the various animal models with inflammation based on the mechanisms of mitochondrial oxidation inhibition and NLRP3 inflammasome activation [15,16,17,18,19,20,21,22,23]. We do not know how H_2_ can inhibit mitochondrial oxidation, as NLRP3 inflammasome activation is poorly understood. In this paper, we hypothesized a possible mechanism by which H_2_ can inhibit NLRP3 activation via the inhibition of mitochondrial oxidation, and then presented a perspective on the potential effects of H_2_ in chronic inflammatory diseases. The medical applications of H_2_ can solve the problems of many chronic inflammation-based diseases including COVID-19. Additionally, H_2_ may even have the potential to control not only inflammatory diseases but also senescence.

## Figures and Tables

**Table 1:** Mechanisms of H_2_ targeting the inhibitions of mtROS production and NLRP3 inflammasome activation.

Desease/Disease models	Species/Cells	Effects of H_2_	Ref. No.
Inflammatory disease including sepsis	RAW264.7	Inhibition of LPS-induced NLRP3 inflammasome activation by targeting mtROS.	15
Acute pancreatitis	Mice	Inhibition of NLRP3 inflammasome activation and decrease in NF-κB activity.	16
Intestinal I/R injury	Rats	Improvement of ischemia/reperfusion injury through NF-κB/NLRP3 pathway.	17
Acute lung injury	Rats	Improvement of limb ischemia/reperfusion-induced lung injury via down-regulating chemerin and NLRP3.	18
Neuropathic pain	Rats	Alleviation of hyperpathia and microglia activation via autophagy mediated NLRP3 inflammasome inactivation.	19
Subarachnoid hemorrhage	Rats	Attenuation of endothelial cell injuries and inhibition of activation of ROS/NLRP3 pathway.	20
Subarachnoid hemorrhage	Rats	Attenuation of subarachnoid hemorrhage-induced early brain injury through inactivation of NF-κB pathway and NLRP3 inflammasomes.	21
Sepsis-associated encephalopathy	Rats	Inhibition of sepsis-associated encephalopathy by Nrf2 mediated NLRP3 pathway.	22
Sepsis	Mice	Amelioration of organ damage and mitochondrial dysfunction via autophagy-mediated NLRP3 inflammasome inactivation.	23
COVID-19	Humans	Potential to inhibit the cascade from NLRP3 to proinflammatory cytokine release and suppress SARS-CoV-2-induced inflammation.	57, 58, 59

mtROS: mitochondrial reactive oxygen species; NLRP3: nucleotide-binding and oligomerization domain-like receptor family pyrin domain-containing 3, LPS: lipopolysaccharide; NF-κB: nuclear factor kappa beta; ROS: reactive oxygen species; Nrf2: NF-E2-related factor 2; I/R: ischemia/reperfusion; COVID-19: coronavirus disease 2019; Ref: References.
